# *Corynebacterium striatum*: A True Pathogen in Chronic Contiguous Osteomyelitis

**DOI:** 10.1155/2024/5020721

**Published:** 2024-02-02

**Authors:** Grishma R. Trivedi, Shehzad S. Merchant

**Affiliations:** ^1^Infectious Diseases Division, University at Buffalo, Buffalo, NY, USA; ^2^Department of Medicine, University at Buffalo, Buffalo, NY, USA; ^3^Buffalo General Medical Center, Buffalo, NY, USA

## Abstract

**Results:**

All patients were cured after successful completion of an antibiotic course with a resolution of infection. Cure was defined as granulation of the infected wound and resolution of clinical symptoms at outpatient follow-up between 6 and 8 weeks.

**Conclusion:**

This series emphasizes that *C. striatum* is often a true pathogen in the setting of CCO. When isolated in polymicrobial infections, a targeted antibiotic therapy towards this pathogen along with other causative pathogens accompanied by surgical intervention is typically required for a successful cure of CCO.

## 1. Introduction

Chronic contiguous osteomyelitis (CCO) is a progressive infection of the bone acquired contiguously through the skin and soft tissue. In young adults, this condition often develops secondary to direct trauma, injury, and fracture, while in the aged, it is commonly seen with underlying diabetes mellitus (DM) and/or vascular insufficiency. The pathogenesis of CCO includes avascular necrosis of bone and formation of sequestrum (dead bone), and therefore, surgical debridement is usually necessary for the cure in addition to targeted antibiotic therapy. A multidisciplinary approach is required for success, including medical and surgical specialties, particularly for complex cases with soft tissue loss [1, 2].

In recent years, CCO incidence has increased presumably due to population aging, increasing prevalence of diabetes and trauma, and improvement of diagnostics [3]. An estimated 11.3% of the US population have diabetes [4]. Over three decades, Kremers et al. reported an increase in the incidence of diabetic foot osteomyelitis (DFO) from 2.3 to 7.6 and an overall increase in the rates of osteomyelitis (OM) from 11.4 to 24.4 per 100,000 person-years [5]. CCO has been associated with increased long-term disability [6] and an increase in mortality [7], higher rates of drug-resistant infection [8], and a substantial financial burden on the health system [3, 9].

CCO is usually polymicrobial with commonly isolated pathogens being *Staphylococcus aureus* (33%), Gram-negative bacilli (20%), Coagulase-negative *Staphylococci* (14%), *Enterococci* (12%), Streptococci (9%), anaerobes (4%), and *Corynebacteria* (4%) [10].

*Corynebacterium* is a genus within the coryneform bacteria. Many nondiphtherial Corynebacterium species are ubiquitous in the environment and commonly colonize the skin and mucous membranes of humans. *Corynebacterium striatum* (*C. striatum*) is one of the lesser-recognized species of Corynebacteria [11]. *C. striatum* is a nonlipophilic, fermentative, Gram-positive bacillus with a prominent striated appearance on Gram stain [12]. *C. striatum* is often considered a colonizer rather a true pathogen in contrast to *C. jeikeium* and *C. minutissimum* [11, 13].

The first case of *C. striatum* infection was published in 1980 in a patient with chronic lymphocytic leukemia who had a pleuropulmonary infection [14]. Lipsky et al. further summarized the role of nondiphtheria Corynebacterium in various clinical infections [15]. Later, Watkins et al. recognized the nature of *C. striatum* as a potential pathogen, especially in immunocompromised hosts or individuals with a foreign body or an indwelling prosthetic device [12]. Over the years, an increasing number of cases of *C. striatum* have been reported, supporting a pathogenic role in causing pleuropulmonary infection [14, 16, 17], bacteremia associated with catheter-related bloodstream infection [18], native [19, 20] and prosthetic [21, 22] valve infective endocarditis, pacemaker-related endocarditis [23], mediastinitis postcardiovascular surgery [24], septicemia [25], and chronic cutaneous infection in a patient with AIDS [26].

Literature on *C. striatum* causing osteomyelitis/bone infections is scarce. Fernández-Ayala et al. (2001) reported a case of lumbar vertebral osteomyelitis due to *C. striatum* [27]. Bhayani et al. have described a review of 13 cases of *C. striatum* bone and joint infections [28], while Boyd et al. pointed out *C. striatum* as an underrecognized pathogen in diabetic foot osteomyelitis [29]. Verma and Kravitz (2016) published a case of a 69-year-old female with advanced rheumatoid arthritis receiving extensive immunosuppressive therapy who developed empyema and osteomyelitis from *C. striatum* [30]. A case series of *C. striatum* as a causative agent of septic arthritis has also been reported by Roy and Ahmad [31]. A systematic review by Mikosavlijevic et al. included 42 studies and 85 individual cases of invasive infections by *C. striatum*, whereby up to 15.3% caused musculoskeletal infections [32]. Recently, the role of this microbe has also been linked to prosthetic joint infections (PJI) [33–35]. Bermejo Olano et al. further elaborated the risk factors associated with diabetic foot osteomyelitis caused by *C. striatum* [36].

The paucity of published data on *C. striatum*-related OM became the basis of this case series.

## 2. Methods

A total of seven patients with CCO were recognized and retrospectively reviewed, and C. striatum was isolated from intraoperative (deep tissues/bone) cultures in a polymicrobial setting. The specimens were inoculated on media (Blood, Chocolate, MacConkey, Columbia Nalidixic acid, Thioglycollate broth) and incubated for 7 days before finalization. All cultures were initially reported as diphtheroids by our institutional clinical microbiology laboratory. Diphtheroids were further identified per request of the Infectious Disease service when a pathogenic role was suspected, by using RapID™ CB PLUS System and verified by matrix-assisted laser desorption ionization-time-of-flight mass spectrometry (MALDI TOF MS, Bruker MALDI Biotyper® CA System, reference library version 2021 claim 6). Antibiotic susceptibility testing could only be performed on the last patient in this series, once susceptibility to this organism became available at our institution through a send-out to ARUP Laboratories (Salt Lake City, Utah, USA) by broth microdilution. Cure was defined as granulation of the infected wound and resolution of clinical symptoms at outpatient follow-up between 4 and 8 weeks.

## 3. Results

### 3.1. Patient 1

A 61-year-old male with HCV-associated cirrhosis (Child-Pugh class A) complicated by unresectable hepatocellular carcinoma (HCC) on Sorafenib over the last month presented initially with MSSA bacteremia, likely secondary to phlebitis from a peripheral venous catheter. Upon evaluation, he was found to have left sternoclavicular joint (SCJ) osteomyelitis based on physical findings and imaging study. He underwent debridement of the left SCJ, wound cultures grew MSSA, and bone biopsy pathology confirmed osteomyelitis. He was managed by a wound vac, six weeks of intravenous antibiotics—initially oxacillin 2 g every 4 hours, then due to elevated transaminases to nafcillin 2 g every 4 hours, and then due to impaired renal function to cefazolin (2 g every 12 hours, and afterwards 2 g every 8 hours once the renal function was improved) for a total of 6 weeks, and two weeks of oral cephalexin 500 mg every 6 hours.

Due to partial healing, persistent discomfort of the surgical site, continued lethargy, and intermittent fevers and chills, the patient was readmitted 4 weeks after completion of the antibiotic course with concern for a wound infection. The patient underwent repeat debridement of the left SCJ. Intraoperative cultures grew a few colonies of *Pseudomonas aeruginosa* (resistant to piperacillin-tazobactam, cefepime, and ciprofloxacin; susceptible to meropenem) and diphtheroids. *Pseudomonas aeruginosa* was thought to be the significant pathogen, and diphtheroids were dismissed as colonizers/contaminants. He was treated with meropenem 1 g every 8 hours.

He initially improved as noted during outpatient clinic follow-up but then developed worsening pain, swelling, and purulent drainage from the left SCJ site while on meropenem. Hence, he underwent another debridement, partial removal of the clavicle, and intraoperative cultures grew diphtheroids as the sole isolate which was speciated to *C. striatum*. The patient was placed on vancomycin 1 g every 12 hours (trough goal 15–20) for six weeks, which resulted in complete granulation of the left SCJ and resolution of clinical symptoms.

### 3.2. Patient 2

A 53-year-old male with diabetes and renal impairment on dialysis underwent a left transmetatarsal amputation (TMA), and intraoperative aerobic and anaerobic cultures grew a few quantities of *E. coli* (resistant to ampicillin, ampicillin-sulbactam, and cefazolin; susceptible to ceftriaxone, cefepime, and ciprofloxacin) and *Enterococcus faecium* and a moderate quantity of diphtheroids. He was treated with ceftriaxone 2 g daily and then switched to cefepime 2 g three times a week to administer after dialysis sessions, and metronidazole for 6 weeks for residual OM at the surgical margin per pathology report of TMA. The diphtheroids were considered colonizers/contaminants. We avoided placing a peripherally inserted central catheter (PICC) for daily ceftriaxone administration in this patient on dialysis as recommended by the nephrologist. We did not use ciprofloxacin due to concern of QTc prolongation and drug-drug interactions. After initial clinical improvement, the patient returned 3 months later with serosanguinous drainage from the dehisced amputation site; OM was evident on the MRI. Cultures from drainage grew rare quantities of *Enterobacter cloacae* (resistant to cefazolin; susceptible to other antibiotics on panel), *Klebsiella oxytoca* (resistant to ampicillin, otherwise susceptible), *Enterococcus faecium* (vancomycin-resistant, VRE), *Staphylococcus lugdunensis* (methicillin-susceptible) and a moderate quantity of diphtheroids. The patient underwent debridement of TMA stump. Diphtheroids grew from intraoperative deep soft tissue aerobic and anaerobic cultures which were identified as *C. striatum*. The patient received a total of 6 weeks of vancomycin (trough 15–20), cefepime 2 g three times a week postdialysis session, and metronidazole, leading to complete granulation.

### 3.3. Patient 3

A 70-year-old female with poorly controlled diabetes complicated by neuropathy was admitted with ketoacidosis. She was found to have a left heel CCO per clinical and MRI findings. She underwent a partial calcanectomy with a synthetic graft. Nonsurgical (on admission, preoperative) and intraoperative bone cultures had polymicrobial growth with *Pseudomonas aeruginosa* and diphtheroids, later identified as *C. striatum*. Upon the patient's preference for oral antibiotics, she was initiated on oral ciprofloxacin and metronidazole (both 500 mg twice daily) ignoring diphtheroids. However, due to the lack of wound granulation on day 12 of antibiotics after surgery, linezolid 600 mg twice daily was added to target the *C. striatum*. It led to a clinical cure after completion of a 4-week course with all three antibiotics. The weekly complete blood count (CBC) was monitored while on antibiotics, which was tolerated well without any side effects being reported.

### 3.4. Patient 4

A 71-year-old male with poorly controlled DM, chronic venous insufficiency, and s/p right BKA presented with a left heel ulcer for which he received outpatient oral levofloxacin 750 mg daily and amoxicillin-clavulanate 875−125 mg twice daily for 2 months without improvement. On admission, the physical exam was consistent with calcaneal OM with a positive probe to bone and visible bone fragments. Superficial wound cultures revealed polymicrobial growth consisting of few quantities of extended-spectrum beta-lactamase (ESBL) *E. coli*, *Staphylococcus simulans*, VRE, and moderate quantities of diphtheroids (and was identified as *C. striatum*). Ertapenem 1 g daily was initiated. However, bone cultures during debridement grew *C. striatum* as a sole organism and started on vancomycin 1 g twice daily with a close-trough monitoring range of 15–20. Both antibiotics were tolerated well with the completion of 6-week course, leading to a complete clinical recovery.

### 3.5. Patient 5

An 87-year-old male with poorly controlled DM presented with a left heel ulcer and was found to have a calcaneal abscess and underlying OM based on clinical exam with a positive probe to bone. Multiple superficial cultures had polymicrobial growth including rare quantities of MRSA, *Enterococcus faecalis* (vancomycin-susceptible), *Morganella* species, and few quantities of diphtheroids. The diphtheroids were identified as *C. striatum*. Initially, the patient received vancomycin 1 g every 12 hours and piperacillin-tazobactam 4.5 g every 6 hours. The patient underwent debridement, and intraoperative deep tissue cultures grew *C. striatum*. The patient was treated with vancomycin (dose around 750 mg daily adjusted based on renal function, to maintain trough level 15) for a total of 4 weeks, targeting *C. striatum* as a pathogen here, with subsequent complete wound granulation.

### 3.6. Patient 6

A 48-year-old female with poorly controlled diabetes complicated by neuropathy, peripheral arterial disease, and coronary artery disease with a prior stent placement was admitted for wet gangrene of the left great toe with a positive probe to bone. He was prescribed cephalexin 500 mg every 8 hours by a primary care physician for a week before this admission. Superficial cultures from drainage grew *Pseudomonas aeruginosa* (pan-susceptible), *E. faecalis* (vancomycin-susceptible MIC 1), and *C. striatum*. She underwent a toe amputation. Intraoperative bone cultures grew *C. striatum* as the sole pathogen. The patient was initially maintained on vancomycin (dosed to maintain trough 15–20) and meropenem 1 g every 8 hours (documented allergy to penicillin) targeting both *C. striatum* and *Pseudomonas aeruginosa*, respectively, and later transitioned to oral linezolid 600 mg twice daily and ciprofloxacin 500 mg twice daily per patient's preference for oral medication for a total of 4 weeks, leading to a complete recovery.

### 3.7. Patient 7

A 61-year-old male with diabetic neuropathy was admitted with a nonhealing right heel ulcer. He had started outpatient oral ciprofloxacin 500 mg twice daily and amoxicillin-clavulanic acid 875−125 mg twice daily, 8 days prior to admission without clinical improvement. He was found to have a calcaneal CCO per clinical and MRI findings. Superficial wound cultures had polymicrobial growth: *Klebsiella oxytoca* (quinolone-susceptible), *Proteus mirabilis*, *Morganella morganii*, *Myroides species*, and moderate diphtheroids. Ciprofloxacin and amoxicillin-clavulanic acid were targeting all isolates except diphtheroids which were thought to be colonizers, though the wound continued to worsen. On admission, the patient was started on piperacillin-tazobactam 4.5 every 6 hours and then after 3 days switched to ceftriaxone 2 g daily and metronidazole 500 mg three times daily. The patient underwent a calcanectomy. Intraoperative bone cultures grew *C. striatum* as a sole pathogen. Vancomycin 1 g every 12 hours for a trough goal 15–20 was added to ceftriaxone and metronidazole for a total of 6-week duration, with subsequent granulation of the wound.

Refer Table 1 for clinical details.

## 4. Discussion

In this case series, we present data that support *C. striatum* as an underappreciated/unrecognized pathogen in the setting of CCO in the absence of foreign material. *C. striatum* is often not identified by the clinical microbiology laboratory but may be reported as diphtheroids. As a result, the clinician may consider it as a colonizer or a contaminant. Therefore, it is critical that diphtheroids be identified and appropriately treated in a timely manner to maximize the chances of cure for this challenging infection. The significance of the treatment of Corynebacterium striatum as a pathogen in the setting of polymicrobial OM was also raised by Roy and Ahmad [31].

*C. striatum* infections are commonly observed in patients with immunocompromised conditions and overall rare in immunocompetent individuals. Martinez-Martinez et al. were amongst the initial authors reporting the clinical significance of *C. striatum* isolation from human samples [37]. However, only 6 out of 26 patients had *C. striatum* isolated from infected leg wounds and there was no clear mention of bone involvement in that retrospective chart review.

Wilson et al. reported a case of chronic osteomyelitis by Corynebacterium species (not further identified) whereby the infection was thought to be of an indolent nature, with the only causative risk factor being an accidental contamination of a postcraniotomy bone flap during surgery 24 years ago [38].

Our experience while treating patient 1 was enlightening in terms of interpreting the role of *C. striatum* as a true pathogen in CCO. We ignored the growth of diphtheroids in multiple cultures, especially when coisolated with *Pseudomonas aeruginosa* until later in the clinical course, when *C. striatum* grew as a sole pathogen. We hypothesize that *C. striatum* was introduced either during the initial surgical intervention or into the postoperative wound. There was incomplete healing of the clavicular wound until targeted antibiotic therapy against *C. striatum* was administered. Sorafenib, an immunosuppressant for HCC, is an unlikely contributor to CCO here, since serious infections are not a recognized complication [39, 40].

While treating patient 2, diphtheroids were disregarded as colonizers/contaminants until the patient returned with a stump infection despite adequate antibiotics against all other pathogens. The diphtheroids were speciated as *C. striatum*, and targeted therapy with vancomycin for 6 weeks led to a complete resolution of infection.

For patients 3 and 4, broad-spectrum antimicrobials did not achieve wound granulation until after focused treatment on *C. striatum* was added.

Learning from our prior experiences, for patients 5 and 6, we recognized *C. striatum* as a pathogen earlier in the clinical course and requested our microbiology lab to identify diphtheroids which were verified as *C. striatum* both pre- and intraoperatively. Both patients had favorable outcomes with complete healing of their wounds after surgical intervention and appropriately targeted antibiotics including against *C. striatum*.

For patient 7, additionally, we were able to obtain a susceptibility result on *C. striatum*. The strain was susceptible to vancomycin, linezolid, and daptomycin while being resistant to tetracycline, beta-lactams, clindamycin, quinolones, and trimethoprim-sulfamethoxazole (TMP-SMX). This result correlates with the published literature regarding *C. striatum* susceptibility.

Vancomycin is the parenteral antibiotic of choice against *C. striatum* due to the recognition of significant resistance to beta-lactam drugs [41]. Although earlier reports indicated *C. striatum* isolates were frequently susceptible to many antimicrobials including beta-lactams, tetracyclines, and fluoroquinolones [37, 41, 42], more recent data have showed an increase in multidrug resistance [43–46]. Also, Milosavlijevic et al. demonstrated that piperacillin-tazobactam and amoxicillin-clavulanic acid may have activity against some strains and therefore should be tested in consideration of treatment [32]. Hahn et al. (University of Washington, Seattle, WA) identified 170 immunocompetent adult patients infected with *C. striatum* and ran susceptibilities to penicillin, ciprofloxacin, clindamycin, erythromycin, and tetracycline on 121/179 isolates, and 72% (87/121) were resistant to all these antimicrobial agents. The only antibiotic that the microbe was uniformly susceptible to was vancomycin with consistent low MICs (mean 0.6, median 0.5, range of 0.125-1) [47]. Additionally, only 10 strains were tested against linezolid, and all were susceptible.

Yamamuro et al. showed 100% susceptibility to vancomycin, linezolid, and minocycline although a high degree of resistance to tetracyclines has been reported previously [37, 41, 42, 47]. Data by Alibi et al. from a Tunisian hospital during 2011–2014 showed all 63 strains of *C. striatum* were susceptible to vancomycin, linezolid, and daptomycin [48]. However, empiric use of daptomycin for the treatment of *C. striatum* may be problematic considering evolving resistance [49, 50], as demonstrated by McMullen et al. whereby rapid *in vitro* development of daptomycin resistance was observed in all tested isolates (*n* = 50) in the study [51]. The clinical impact of this is well described by Streifel and colleagues in a case report of worsening PJI while on daptomycin, demonstrating MIC creep (0.064 to >256 *μ*g/mL via E-test), requiring another surgery and a change of antibiotic to linezolid [52]. Dalbavancin can be considered as another therapeutic option against *C. striatum* which has shown a favorable outcome at >1 year in a patient with PJI requiring long-term therapy and encountered side effects with previously administered agents (vancomycin, linezolid, daptomycin), as reported by Söderquist et al. [34]. Hence, while summarizing the susceptibilities of *C. striatum*, it appears that vancomycin and linezolid are the only reliable antibiotics which have shown consistently uniform susceptibility and other antibiotics should be tested before being considered as a treatment agent.

Limitations of this report include it being a retrospective case series. Although the role of *C. striatum* as an offending pathogen was strong in these cases based on the clinical course and culture data, the relative importance of surgical debridement versus antimicrobial therapy can be difficult to decipher. Nonetheless, a combined surgical-medical approach will maximize the chances of cure since it is often difficult to determine if and to what degree residual osteomyelitis is present after debridement/resection. Patients were followed until granulation of the wound was achieved. Hence, a late relapse cannot be excluded. Lastly, a potential limitation for the management of *C. striatum* infection is a lack of access to susceptibility testing. However, in this series, the lack of initial availability of susceptibility data did not affect the patient outcomes since empirical use of vancomycin and linezolid is predicted to be active, as evidenced by the complete recovery of our patients.

## 5. Conclusion

Timely recognition of *C. striatum* as a pathogen is crucial in the management of CCO. Data from this report supports the potential importance of *C. striatum* in the pathogenesis of CCO. The identification of diphtheroids on culture should prompt speciation, and if *C. striatum* is identified, consideration for treatment should be given to maximize the chances for cure.

## Figures and Tables

**Table 1:** Patients' characteristics.

Patients	Age/sex	Comorbities	OM location	Diagnosed by	Surgical intervention	Microbiology				Outcome^a^
						Preoperative cultures	Prior antibiotics	Intraoperative cultures	Antibiotics postintervention	
1	61/M	Unresectable HCC (on sorafenib), cirrhosis due to HCV	Left sternoclavicular joint	Positive PTB	Debridement, clavicle removal	MSSA, *P. aeruginosa*, diphtheroids	(Nafcillin, cefazolin, cephalexin), meropenem	*C. striatum*	Vancomycin	Cure
2	53/M	IDDM, neuropathy, ESRD via A-V fistula, s/p left TMA	Left TMA amputation	Positive PTB, MRI	Debridement	*E. coli*, *Enterococcus faecium*, diphtheroids	Ceftriaxone- > cefepime, metronidazole	*Enterobacter cloacae*, *K. oxytoca*, *Enterococcus faecium* (VRE), *Staph. lugdunensis*, *C. striatum*	Vancomycin, cefepime, metronidazole	Cure
3	70/F	IDDM, polyneuropathy, CAD, CHF, and tobacco abuse	Left calcaneus	Positive PTB, MRI	Partial calcanectomy with graft	*P. aeruginosa*, diphtheroids	Ciprofloxacin, metronidazole	*C. striatum*, VRE	Linezolid, ciprofloxacin, metronidazole	Cure
4	71/M	DM, s/p R BKA, chronic venous stasis	Left heel	Positive PTB	Debridement	Polymicrobial, not identified further	Amoxicillin-clavulanate, levofloxacin	*C. striatum* ESBL *E.coli* VRE	Vancomycin, ertapenem	Cure
5	87/M	DM, CHF, prostate cancer in remission	Left calcaneus	Positive PTB	Debridement	*E. faecalis*, MRSA, Morganella spp, *C. striatum*	Vancomycin, piperacillin-tazobactum	*C. striatum*	Vancomycin	Cure
6	48/F	Diabetic neuropathy, PAD, and tobacco abuse	Left great toe	Positive PTB	Amputation	*P. aeruginosa, E. faecalis*, *C. striatum*	Cephalexin- > vancomycin, meropenem	*C. striatum*	Vancomycin, meropenem- > linezolid, ciprofloxacin	Cure
7	61/M	DM neuropathy	Right calcaneus	Positive PTB, MRI	Excisional debridement, calcanectomy	*K. oxytoca*, *Proteus mirabilis*, *Myroides spp*, diphtheroids	Amoxicillin-clavulanate, ciprofloxacin	*C. striatum*	Vancomycin ceftriaxone metronidazole	Cure

^a^Cure is defined as granulation of the infected wound and resolution of clinical symptoms at 6- to 8-week follow-up. A-V fistula: arteriovenous fistula; CAD: coronary artery disease; CHF: congestive heart failure; *C. striatum*: *Corynebacterium striatum*; DM: diabetes mellitus; *E. faecalis*: *Enterococcus faecalis*; *E.coli*: Escherichia coli; ESBL: extended-spectrum beta-lactamase; ESRD: end-stage renal disease; HCC: hepatocellular carcinoma; HCV: hepatitis C virus; *K. oxytoca*: *Klebsiella oxytoca*; MSSA: methicillin-susceptible *Staphylococcus aureus*; MRI: magnetic resonance imaging; MRSA: methicillin-resistant *Staphylococcus aureus*; OM: osteomyelitis; PTB: probe to bone; Staph: *Staphylococcus*; VRE: vancomycin-resistant *Enterococcus*; *P. aeruginosa*: *Pseudomonas aeruginosa*; TMA: transmetatarsal amputation.

## Data Availability

Data are available as a part of this manuscript.

## Abbreviations

AIDS: Acquired immunodeficiency syndrome
BKA: Below knee amputation
CBC: Complete blood count
CCO: Chronic contiguous osteomyelitis
*C. striatum*: *Corynebacterium striatum*
DFO: Diabetic foot osteomyelitis
DM: Diabetes mellitus
ESBL: Extended-spectrum beta-lactamase
HCV: Hepatitis C virus
HCC: Hepatocellular carcinoma
MALDI TOF MS: Matrix-assisted laser desorption ionization-time-of-flight mass spectrometry
MSSA: Methicillin-susceptible *Staphylococcus aureus*
OM: Osteomyelitis
PJI: Prosthetic joint infection
SCJ: Sternoclavicular joint
TMA: Transmetatarsal amputation
TMP-SMX: Trimethoprim-sulfamethoxazole.
